# A Treatise on Medical Jurisprudence

**Published:** 1856-03

**Authors:** 


ART. VI.—*A Treatise on Medical Jurisprudence.* By FRANCIS WHARTON, author of a "Treatise on American Criminal Law," "Precedents of Indictments," "American Law of Homicide," etc. And MORETON STILLE, M. D., Lecturer on the Principles and Practice of Medicine in the Philadelphia Association for Medical Instruction. Philadelphia: Kay and Brother. 1855.

The first emotion with which the reviewer greets this volume, is one of regret that Dr. Stillé should not have lived to reap the reputation it was sure to bestow upon him. Wearied by his labors, he repaired to Saratoga, after their completion, for rest and recovery, and there suddenly found rest from every labor in the quiet of the grave. But dying as he did thus early, his earnest and ambitious spirit could hardly have desired a more enduring and worthy monument than his own share of this Treatise on Medical Jurisprudence.

A new volume on this subject was not a work of supererogation. Beck's Treatise, though it had attained an unexampled popularity in the profession, and had reaped the honors of several reprints and translations in England and on the Continent, was deficient, in that it was the production of a medical man only. While to the physician it appeared everything that could be desired, and while the legal profession cheerfully accorded to it the position of a standard authority, yet there was among lawyers a feeling of dissatisfaction at its *legal* deficiencies, and an earnest demand for a work which, while it possessed the vital element of correct medical reasoning, should also possess an indisputable legal authority.

We suppose this desideratum to have been obtained in the book under notice. The reviewer, familiar with the well earned reputation of the two brothers Stillé, cannot hesitate to accord to it a high standard of medical merit, and while we are, from mere accident of association, unfamiliar with the legal position of Mr. Wharton, we are willing to take it for granted that Dr. Stillé would have associated with himself no inferior talent. We may trust, then, that the object so earnestly desired by the legal profession has been attained, and that we have now a work, standard in both departments.

In our advancing civilization, when people die as often by foul means as fair; when skillful toxicologists insure the lives of their victims before administering the insidious poison which will slowly, but surely, destroy them; when "intra-uterine murder" is so fashionable a crime that the most high-minded physician is every day liable to the insult of a proposition to commit it—that proposition coming more often from the soft-voiced and gently-nurtured married lady, who fears the effect of a pregnancy upon her capacities for dancing; who assures him that her figure will be ruined by parturition, or that she would not mind it if the disagreeable event could only happen in the season of Lent, than from the poor victim of the seducer, whose wretched situation is a real and almost irresistible temptation to crime—when the false philosophies of phrenology, spiritualism, and free-love, have created equally false distinctions in the nature of crime, and would permit the individual to govern his actions by a "higher law" than the Decalogue, would add codicils to the commandments, and make the seventh to read, "Thou shalt not commit adultery—except thou hast a passional attraction!" and the eighth, "Thou shalt do no murder—except thou can'st mimic insanity!" it is time that the two great and learned professions which stand as barriers to the inroads of wickedness, should understand each other, and act together in its detection and punishment.

We do not intend a *review* of Wharton and Stillé's Medical Jurisprudence. Asking our readers to take our word for it that it is a valuable work, we wish to devote the little space we have to a brief exhortation on the importance of a thorough and competent knowledge of this much neglected branch of medical science, to the ordinary practitioner.

The medical witness is, in important cases, usually pitted against the highest legal talent; and, as we have sometimes blushed to notice, the lawyer may sometimes come to the trial of his cause with a more thorough knowledge of the anatomical or surgical fact in question, than the witness himself. In a case of malpractice occurring in our Supreme Court some eighteen months since, a practitioner of four or five years' standing, testified that in that brief period he had reduced *five* dislocations of the wrist joint! more than are recorded in the annals of surgery! Lawyers sat by and listened to this evidence, who knew that the accident was so rare that its existence was doubted. What an exalted opinion must they have had of medical testimony!

On the contrary, we have known a young physician called to testify to post-mortem appearances in a case of suspected murder. The evidence turned on the intricate questions of sugillation and ecchymosis, and the counsel for the defence—one of the highest ornaments of the bar in western New York—selected this witness as a proper subject for "badgering," a conclusion well warranted by the sleepy, fat, and good-natured aspect of the young physician. But he mistook his man; for once roused by the browbeating of the counsel, he showed such a thorough knowledge of the subject in all its bearings, that the eyes of hundreds of spectators were for the first time opened to the learning, talent, and faculties of observation concealed beneath the quiet manner of that young witness.

In cases of criminal jurisprudence especially, it is in the power of the medical witness to do the highest honor to his profession, or to disgrace it and himself to an equal extent. The subject, however, is one neglected in our schools, and unstudied by the practitioner, absorbed, as he is, in his everyday labors. Very often great injustice is done to the real worth of the medical man, from his ignorance of questions for which he has never seen cause to prepare himself. But for this he is himself at fault. Every man is liable to be called upon to support his own credit and that of the profession in the witness box. And he should reflect, that, aside from this selfish motive, he should regard the momentous interests which may rest upon his word. An infant is found in a hidden place. Was it still-born, or had it lived? Was its mother comparatively innocent, or was she a murderess? A married couple are found dead together in their room, say from the fumes of charcoal. The heirship to an estate depends upon which died first. Upon which of the two does the presumption of survivorship rest? A corpse is found with deadly wounds inflicted upon it. Is this a suicide, or but the mockery of one? And so on, a thousand questions may arise which call for all the learning, acuteness, and observing powers of the shrewdest mind.

It may be said that we are recounting stale truths. But if so they will bear repetition so long as physicians rush to the witness-box to decide the most important questions without study or preparation, and to lay themselves and their profession open to the sneers of the well-informed by their ignorance. The solemn owlishness of manner which so easily captivates the feeble wits of the old ladies of his neighborhood, is but a sorry protection against the worrying cross-examination of the keen and subtle lawyer, who has spent a week in preparation for that single interview.

For sale by WANZER, MCKIM & Co.

